# Synthesis of Some Phenylpropanoid Glycosides (PPGs) and Their Acetylcholinesterase/Xanthine Oxidase Inhibitory Activities 

**DOI:** 10.3390/molecules16053580

**Published:** 2011-04-28

**Authors:** Xiao-Dong Li, Shuai-Tao Kang, Guo-Yu Li, Xian Li, Jin-Hui Wang

**Affiliations:** 1 School of Traditional Chinese Materia Medica, Shenyang Pharmaceutical University, Shenyang 110016, China; Email: lxdpharm@sina.com (X.-D. L.); 2 School of Pharmacy, Shihezi University, Shihezi 832002, China; 3 Key Laboratory of Phytomedicine Resources & Modernization of TCM of Ministry of Education, Shihezi 832002, China; 4 Key Laboratory of Structure-Based Drug Design & Discovery of Ministry of Education, Shenyang Pharmaceutical University, Shenyang 110016, China

**Keywords:** phenylpropanoid glycosides, *Rhodiola rosea* L., acetylcholinesterase/xanthine oxidase inhibitory activity

## Abstract

In this research, three categories of phenylpropanoid glycosides (PPGs) were designed and synthesized with PPGs isolated from *Rhodiola rosea* L. as lead compounds. Their inhibitory abilities toward acetylcholinesterase (AChE) and xanthine oxidase (XOD) were also tested. Some of the synthetic PPGs exhibited excellent enzyme inhibitory abilities.

## 1. Introduction

**Figure 1:**
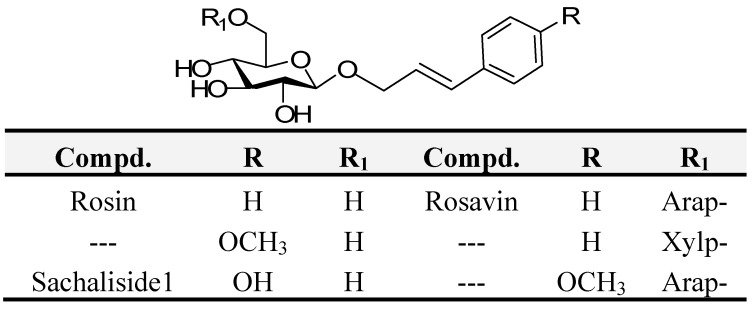
Phenylpropanoid glycosides Isolated from *Rhodiola rosea L.*

*Rhodiola rosea* L. has been used in Chinese herbal medicine to stimulate the nervous system, decrease depression, enhance work performance, resist anoxia, eliminate fatigue, prevent high altitude sickness and improve sleep, *etc*. Clinical studies show that *Rhodiola rosea* extract has the ability to improve mental ability and learning behavior [1,2,3]. Recent literature indicates that it can improve resistance to cerebral ischemia and reduce myocardial infarction area. It also has therapeutical effects on coronary heart disease and hyperlipidemia [4,5,6,7]. Most of the reported activities were confined to the plant itself or its extract. Many trace components such as amino acids, polysaccharides, steroids and phenylpropanoid glycosides have been isolated from *Rhodiola rosea* L. and phenylpropanoid glycosides (Figure 1) are considered to be the major active components. However, the low content of PPGs in this plant has limited the further investigation of their activities.

Cholinesterase inhibitors are the only currently approved drugs for treating patients with mild to moderately severe Alzheimer’s disease, a disorder associated with progressive degeneration of memory and cognitive function. The cholinergic hypothesis postulates that memory impairment in patients with Alzheimer’s disease result from a deficit of cholinergic function in the brain [8,9]. The most important changes observed in the brain are a decrease in hippocampal and cortical levels of the neurotransmitter acetylcholine and associated enzyme choline transferase. 

Acetylcholinesterase inhibitors can restore the level of acetylcholine by inhibiting acetylcholinesterase. Xanthine oxidase (XO) is a key enzyme in the purine metabolic pathway, catalyzing the oxidation of hypoxanthine to xanthine, and then to uric acid [10]. Xanthine oxidase and xanthine dehydrogenase (XDH) are the isomers of xanthine oxidoreductase (XOR). XO receives molecular oxygen resulting in superoxide anion (O_2_^−^) which causes serial harmful damages to vascular endothelial cell. The rise of XO protein level has been observed in heart failure models of both animal and human, which plays an important role in pathophysiological process of heart failure [11,12,13,14,15]. 

In order to provide the pharmacological basis for the usage of *Rhodiola rosea* L. in the therapy of nervous and cardiovascular diseases in Traditional Chinese Medicine, it was deemed necessary to synthesize these PPGs and evaluate their inhibitory activities towards acetylcholinesterase (AChE) as well as xanthine oxidase (XOD).

## 2. Results and Discussion

### 2.1. Chemistry

**Scheme 1:**
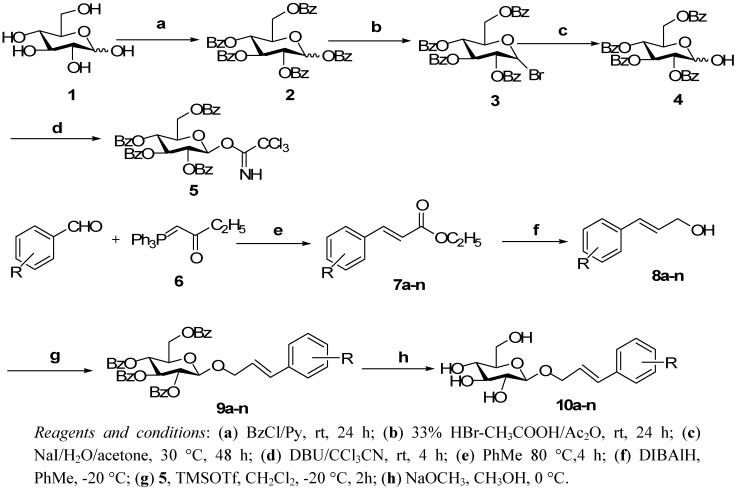
The synthetic route of the target PPGs **10a-n**

The designed synthetic route of these PPGs is depicted in Scheme 1. Compound **4** was prepared *via* benzoylation of D-glucose followed by 1-*O*-bromination and sequential regioselective C-1 hydrolization with NaI as catalyst in a mixed solution of acetone-water (3:1). Treatment of the hemiacetal **4** with 1,8-diazabicyclo[5,4,0]undec-7-ene (DBU) and excess trichloroacetonitrile furnished the corresponding trichloroacetimidate donor **5**. 

Condensation of different benzaldehyde derivatives with [(ethoxycarbonyl)methylene]triphenyl-phosphorane (**6**) afforded the target ethyl esters **7a**-**n** in satisfactory yields. The latter were next reduced to the corresponding alcohol derivatives **8a**-**n** using DIBAlH in toluene. Treatment of **8a**-**n** with excess trichloroacetimidate under the influence of catalytic amounts of TMSOTf in CH_2_Cl_2_ at −20 °C to room temperature for 2 h afforded the tetrabenzoyl PPGs derivates **9a-n**. Finally, the protective groups were readily removed using NaOMe in MeOH to afford the desired PPGs **10a-n**.

**Scheme 2:**
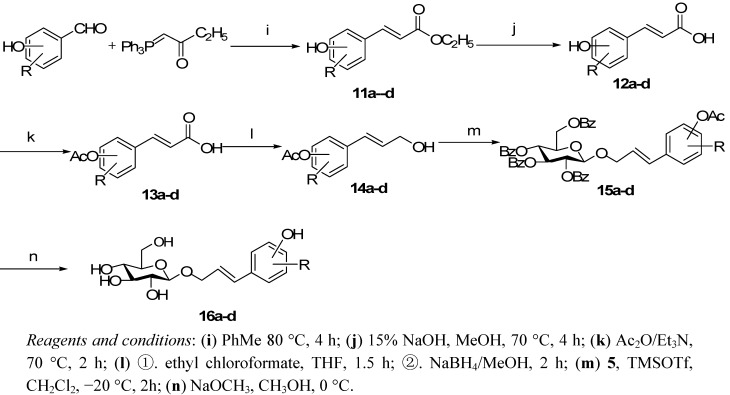
The preparation of the target PPGs **16a-d**.

In order to introduce the hydroxyl group onto the aromatic rings an alternative route was chosen and is depicted in Scheme 2. Coupling of benzaldehyde derivates with the same ylide reagent **6** gave compounds **11a**-**d**, which were then hydrolyzed and acetylated to produce **13a**-**d**, which in turn were treated with ethyl chloroformate in THF to form the corresponding anhydride intermediates. Reduction of the latter by adding sodium borohydride and a calculated amount of anhydrous methanol to the above solutions afforded the desired alcohols **14a**-**d**, which were then glycosidated and deacylated using conditions similar to those used for **10a**-**n** and we thus obtained **16a**-**d**.

**Scheme 3:**
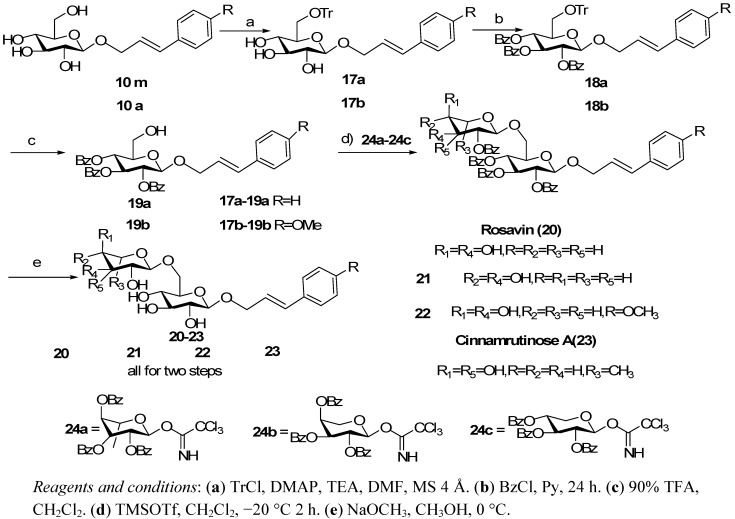
The synthetic approaches to the target disaccharide PPGs.

The designed synthetic route for disaccharide PPGs was as shown in Scheme 3. Compounds **24a-c **were prepared *via* a similar method to that used in the preparation of compound **5** [16]. Reaction of trityl chloride with **10m** or **10a** in *N,N*-dimethylformamide (DMF) solution for 36 h at room temperature in the presence of 4-*N,N*-dimethylaminopyridine (DMAP), powdered 4 Å molecular sieves and triethylamine cleanly produced **17a** or **17b** in high yields (56% and 64%, respectively) [17]. Compounds **17a** and **17b** were then converted to their tribenzoyl derivatives **18a** and **18b** by using benzoyl chloride in pyridine. 

Compounds **18a** and **18b** were then treated with 90% aqueous TFA to selectively remove the trityl group and we thus acquired the key intermediates **19a** and**19b**. Treatment of **19a** and **19b** with excess trichloroacetimidates **24a-c **under the influence of catalytic TMSOTf in CH_2_Cl_2_ at −20 °C to room temperature for 2 h afforded the corresponding hexabenzoyl derivatives. Finally, treatment of the coupling products with NaOMe in MeOH/CH_2_Cl_2_ provided the synthetic targets rosavin (**20**), cinnamyl 6-*O*-(β-D-xylopyranosyl)-β-D-glucopyranoside (**21**), 4-methoxycinnamyl 6-*O*-(α-L-arabino-pyranosyl)-β-D-glucopyranoside (**22**) and cinnamyl 6-*O*-(α-L-rhamnopyranosyl)-β-D-glucopyranoside (**23**). The spectral data (^1^H- and ^13^C-NMR) and specific rotation of the synthetic PPGs were consistent with those of the natural products reported in the literature [18,19,20].

### 2.2. AChE and XOD Inhibitory Activities of the Synthetic PPGs

*In vitro* inhibitory activity test of these glycosides against AChE and XOD was studied by using Acetylcholinesterase Detection Kit and Xanthine Oxidase Detection Kit. The results were summarized and shown in Table 1. 

**Table 1:** Effect of the synthetic PPGs on acetylcholinesterase and xanthine oxidase

Compd.	Structure		AChE inhibitory activitiy		XOD inhibitory activity	
	R_1_	R	% inhibition (1.5 mg/mL)	IC50 (μM) ^a^	% inhibition (1.5 mg/mL)	IC50 (μM) ^a^
**10a ^I^**	H	4-OCH_3_	15 ± 3.27	–	10 ± 2.22	–
**10b**	H	2-OCH_3_	62 ± 4.53	15.5 ± 2.1	12 ± 3.12	–
**10c**	H	3,5-di-OCH_3_	23 ± 0.85	–	21 ± 1.21	–
**10d ^I^**	H	3,4-di-OCH_3_	43 ± 1.35	43.5 ± 0.6	35 ± 3.22	73.5 ± 1.5
**10e**	H	2,3-di-OCH_3_	34 ± 4.36	71.2 ± 2.8	22 ± 0.85	–
**10f**	H	4-CF_3_	22 ± 2.44	–	5 ± 0.22	–
**10g**	H	3,4(-OCH_2_O-)	15 ± 3.55	–	13 ± 1.43	–
**10h ^I^**	H	3,4,5-tri-OCH_3_	17 ± 4.33	–	33 ± 2.16	69.3 ± 1.3
**10i**	H	2-F	18 ± 2.11	–	24 ± 1.32	–
**10j**	H	4-Cl	42 ± 3.23	38.3 ± 1.4	50 ± 2.35	24.3 ± 1.4
**10k**	H	3,4-di-F	43 ± 2.31	35.5 ± 1.1	37 ± 2.78	55.4 ± 1.3
**10l**	H	4-Br	57 ± 1.22	22.4 ± 0.6	55 ± 3.67	19.6 ± 1.5
**10m ^I^**	H	H	16 ± 0.44	–	38 ± 2.83	93.2 ± 1.4
**10n**	H	3-Br,4-OC_2_H_5_, 5-OCH_3_	36 ± 3.55	87.3 ± 2.1	28 ± 1.74	97.3 ± 0.8
**16a ^I^**	H	4-OH	34 ± 2.67	75.2 ± 1.2	32 ± 1.63	79.3 ± 0.7
**16b**	H	3-OH	75 ± 2.12	7.62 ± 1.1	41 ± 2.22	29.6 ± 1.2
**16c ^I^**	H	3-OCH_3_,4-OH	54 ± 2,34	25.7 ± 0.7	23 ± 0.93	–
**16d**	H	3-OH,4OCH_3_	66 ± 3.45	10.3 ± 2.0	56 ± 2.34	16.5 ± 1.1
**20 ^I^**	α-L- Ara*p*-	H	85 ± 0.21	1.72 ± 0.1	73± 0.02	5.55 ± 0.0
**21 ^I^**	β-D- Xyl*p*-	H	82 ± 0.11	3.71 ± 0.1	74± 0.12	4.56 ± 0.1
**22 ^I^**	α-L- Ara*p*-	4-OCH_3_	72 ± 0.10	4.23 ± 0.1	65± 0.10	5.71 ± 0.1
**23 ^I^**	α-L- Rha*p*-	H	81 ± 0.05	2.05 ± 0.0	60± 0.01	8.21 ± 0.0

^a^ Data are means ± standard deviation of triplicate independent experiments. –, not active (less than 30% inhibition at 1.5 mg/mL). ^I ^natural products.

The data listed in Table 1 clearly show that most of the designed compounds such as **10b**, **10l**, **16b-d** and compounds **20-23 **exhibited moderate inhibitory activities toward cholinesterase. In the synthetic monosaccharide PPGs, compounds with sterically small substituents at position 4 (compounds **10b**, **10j**-**l**) were more potent than the corresponding unsubstituted compound **10m**. The presence of a hydroxyl group at the aromatic ring caused a significant improvement in the activity. Disaccharide glycosides always possessed much stronger AChE inhibitory activities than the corresponding momosaccharide PPGs, with rosavin being the most potent (IC_50_ = 1.72 μmol/L). Accordingly, the XOD inhibitory activity of these PPGs was generally lower than that against AChE. Compounds **10j**, **10l**, **16b**, **16d **and **20-23** have XOD inhibitory activity. Among them compound **21 **showed the best such activity, with an IC_50_ value of 4.56 μmol/L. The above results indicate that disaccharide glycosides and monosaccharide PPGs with a substitution of sterical small group at position 4 or hydroxyl at the aromatic ring are favorable for the enzymes inhibitory activities which are worthy of further study.

## 3. Experimental

### 3.1. General

Commercial reagents were used without further purification unless otherwise stated. The boiling range of petroleum ether was 60–90 °C. Preparative TLC was done on silica gel plates (GF254; Qingdao Haiyang Co.; China). Preparative column chromatography was performed with silica gel (200–300 mesh; supplier as above). Melting points were measured with a Yanaco apparatus and are uncorrected. NMR spectra were recorded on Bruker ARX 300 MHz or AV 600 MHz spectrometers; *J* values were given in Hertz, *δ* in ppm rel. to TMS used as internal standard. Optical rotations were measured at the sodium D-line at room temperature with a Perkin-Elmer 241 MC polarimeter. ESI mass spectra were obtained on a Finnigan TSQ 7000 mass spectrometer. High-resolution mass spectra (HR-ESI-MS) were obtained with Bruker micro TOF-Q 125 mass spectrometer.

*2,3,4,6-Tetra-O-benzoyl-β-D-glucopyranose* (**4**). D-Glucose (10.0 g, 55.5 mmol) in pyridine (150 mL) was cooled to 0 °C, and then benzoyl chloride (38.5 mL, 333 mmol) was added dropwise over 30 min. The reaction mixture was raised to room temperature. After 24 h, water (100 mL) was added to the reaction mixture, and stirring was continued for 30 min. The aq solution was extracted with CH_2_Cl_2 _(3 × 150 mL). The extract was washed with HCl (1 N) followed by saturated sodium bicarbonate (2 × 150 mL), dried (Na_2_SO_4_) and concentrated to dryness to give a yellow solid which was directly dissolved in a solution of anhydrous dichlormethane (230 mL) containing 33% HBr in glacial acetic acid (50 mL) and acetic anhydride (7 mL). The reaction mixture was stirred for 24 h at 34 °C, and then ice-cold water (200 mL) was added. The mixture was extracted with CH_2_Cl_2_ (3 × 100 mL). The combined CH_2_Cl_2_ layers were washed with a saturated NaHCO_3_ solution and brine, and the filtrate was evaporated in vacuo to give a colorless white solid. The white solid was dissolved in acetone (144 mL) and water (48 mL). After addition of sodium iodide dihydrate (2.22 g, 7.5 mmol) the mixture was stirred at 30°C for 2–3 days before removing the solvents *in vacuo*. The residue was resuspended in water (100 mL) and the resulting water phase was extracted with dichloromethane (3 × 100 mL). The combined organic layers were washed with 10% sodium thiosulfate, saturated sodium bicarbonate and water, dried (Na_2_SO_4_). The filtrate was concentrated *in vacuo* to give the crude product (28.0 g, 47 mmol) as a white solid (85% yield for the three steps), m.p. 65–68 °C. ^1^H-NMR (600 MHz, CDCl_3_): δ 8.07–7.30 (20H, m, Ar-H × 20), 6.06 (1H, t, *J* = 10.2 Hz, CH), 5.62 (1H, t, *J* = 10.2 Hz, CH), 5.53 (1H, t, *J* = 3.6 Hz, CH), 5.20 (1H, d, *J* = 7.2 Hz, CH), 4.62 (1H, d, *J* = 10.0 Hz, CH), 4.48 (1H, d, *J* = 12.6 Hz, CH), 4.42 (1H, dd, *J* = 12.0, 4.2 Hz, CH). HRMS (ESI-TOF) calcd. for C_34_H_28_O_10_Na (M+Na)^+ ^619.1582 found 619.1580.

*2,3,4,6-Tetra-O-benzoyl-β-D-glucopyranosyl tricholoroacetimidate*(**5**). The above product (19 g, 31.9 mmol) and trichloroacetonitrile (13.7 g, 95 mmol) were dissolved in CH_2_Cl_2_ (100 mL) and a catalytic amount of DBU (0.48 mL) was added. The above solution was stirred for four hours at room temperature, then concentrated and purified by Al_2_O_3_ column chromatography with pet.ether/EtOAc (3:1) as eluent to give the product (20.0 g, 27.1 mmol) as a foamy white solid in 80% yield. ^1^H-NMR (600 MHz, CDCl_3_): δ 8.63 (s, 1H, NH), 8.10-7.87 (8H, m, Ar-H ×8), 7.58–7.30 (12H, m, Ar-H × 15), 6.80 (d, 1H, *J*_1,2_ = 3.6 Hz, H-1), 6.26 (t, 1H, *J*_2,3_ = *J*_3,5_ = 10.2 Hz, H-3), 5.82 (t, 1H, *J*_3,4_ = *J*_4,5 _= 10.2 Hz, H-4), 4.69 (dd, 1H, *J*_1,2_ = 4.2 Hz, *J*_2,3 _= 9.6 Hz, H-2), 4.65, 4.64, 4.49 (3H, m, H-5, H-6, H-6′). ^13^C-NMR (75 MHz, DMSO-*d_6_*): δ 165.4 (PhCO-), 165.2 (PhCO-), 164.7 (PhCO-), 166.6 (PhCO-), 163.0 (C=NH), 133.8 (Ar-C), 133.7 (Ar-C), 133.6 (Ar-C), 133.5 (Ar-C), 129.3 (8C, 8Ar-C), 128.8 (12C, 12Ar-C), 93.1 (C-1), 89.5 (CCl_3_), 73.4(C-2), 70.5 (C-4), 68.2 (C-3), 66.7 (C-5), 62.4 (C-6). HRMS (ESI-TOF) calcd. for C_36_H_28_Cl_3_NO_10_Na (M+Na)^+ ^762.0676 found 762.0679.

*General procedure for preparation of compounds*** 8**. A mixture of the appropriate benzaldehyde derivative (5 mmol) in anhydrous toluene (40 mL) and (carbethoxymethylene)triphenyl-phosphorane (1.99 g, 6 mol) was refluxed for 4 h. The reaction was monitored by TLC using petroleum-ethyl acetate (3:1) as the mobile phase. The solvent was removed and the residue was suspended in water and the resulting water phase was extracted with ethyl acetate. The combined organic layers were dried (Na_2_SO_4_) and filtered. The solvent was concentrated then purified by column chromatography with pet.ether/EtOAc (3:1) to give compounds **7** which were then dissolved in CH_2_Cl_2_. Three equivalents of DIBAlH in toluene were added dropwise to the above solution at −20 °C. After 2 h, water was added and the mixture solution was extracted with EtOAc, concentrated. The crude products were purified with a short silica gel column to afford compounds **8**. 

*General procedure for preparation of compounds*** 14**. 15% NaOH solution (20 mL) was added to the solution of compounds **11** (5 mmol) in MeOH. The mixture was refluxed for 3 h until the yellow oil disappeared and then cooled to room temperature. The organic solvent was removed. The residue was diluted with water and acidified by 10 N HCl to pH 1 to afford a white precipitate. The precipitate was collected and recrystallized from anhydrous ethanol, affording white needle-like crystals **12**. To a 25 mL flask equipped with CaCl_2_ drying tube, phenol **12**, acetic anhydride (10 equiv) and triethylamine (2.0 equiv) were added. The mixture was refluxed at 70 °C for 4 h and water was added. The above solution was stirred until it became turbid. The precipitate was collected, washed with water and dried to give the product **13**. To a solution of dry THF containing compounds **13** and triethylamine (1.1 equiv) was added dropwise ethyl chloroformate (1.1 equivalents) at 0 °C. After stirring for an additional 30 min, the reaction mixture was allowed to warm to r t and 4.0 equiv. of powdered sodium borohydride was added in one portion. To this suspension, 16 equiv. of absolute methanol was added dropwise over one hour at the same temperature. After being stirred at room temperature for an additional one hour, the reaction mixture was poured into saturated ammonium chloride solution (150 mL) and extracted with dichloromethane (2 × 150 mL). The combined organic layer were dried over anhydrous Na_2_SO_4_ and concentrated. Crude products were purified by silica gel column chromatography (pet. ether/EtOAc 3/1) to give the products **14**.

*Schmidt’s reaction to prepare the target PPGs* **10a**-**n** *and* **16a**-**d**. Alcohols (1.5 mmol), the Schmidt donor **5 **(6.0 mmol, 1.2 equiv), and powdered 4 Å molecular sieves (1.0 g) in dry CH_2_Cl_2_ (20 mL) were stirred for 30 min at −20 °C. A dry CH_2_Cl_2_ solution (0.5 mL) of TMSOTf (0.02 equiv) was added dropwise. The mixture was stirred for 1 h before a small amount of Et_3_N was added to quench the reaction. The mixture was then diluted with CH_2_Cl_2_ and filtered. The resulting residue was dissolved in dry CH_2_Cl_2_-MeOH (1:2), to which NaOMe (4.0 equiv) was added. The solution was stirred at rt for 2 h and then neutralized with Dowex H^+ ^resin to pH 7 and filtered. The filtrate was concentrated and purified with a silica gel column to give the products as white powder.

*4-Methoxycinnamyl β-D-glucopyranoside* (**10a**). Yield: 41%. [*α*]^25^_D_= –35.9 (*c* = 1.0, MeOH). ^1^H-NMR (300 MHz, methanol-*d_6_*): *δ* 7.34 (d, 2H, *J* = 8.7 Hz, Ar-H), 6.85 (d, 2H, *J* = 7.9 Hz, Ar-H), 6.60 (d, 1H, *J* = 15.9 Hz, =CH), 6.21 (dt, 1H, *J* = 6.6, 15.9 Hz, =CH-CH_2_), 4.50 (dd, 1H, *J* = 5.8, 12.4 Hz, =CH-CH_2_), 4.36 (d, 1H, *J* = 7.7 Hz, H-1), 4.29 (dd, 1H, *J* = 6.8, 12.4 Hz), 3.87 (d, 1H, *J* = 11.7 Hz), 3.78 (s, 3H, -OCH_3_),3.67 (dd, 1H, *J* = 5.2, 11.9 Hz, =CH-CH_2_), 3.35–3.18 (m, 4H). HRMS (ESI-TOF) calcd. for C_17_H_23_O_9 _(M+HCOO)^–^ 371.1342; found 371.1342.

*2-Methoxycinnamyl β-D-glucopyranoside* (**10b**). Yield: 45%. [*α*]^25^_D_= –25.5 (*c* = 1.0, MeOH). ^1^H-NMR (300 MHz, methanol-*d_6_*): *δ* 7.45 (d, 1H, *J* = 7.8 Hz, Ar-H), 7.22 (t, 1H, *J* = 7.7 Hz, Ar-H), 6.94 (d, 1H, *J* = 15.6 Hz, =CH), 6.93 (d, 1H, *J* = 7.8 Hz, =CH), 6.90 (t, 1H, *J* = 6.6 Hz, =CH), 6.36 (dt, 1H, *J* = 6.3, 16.2 Hz, =CH-CH_2_), 4.53 (dd, 1H, *J* = 5.7, 13.5 Hz, =CH-CH_2_), 4.40 (d, 1H, *J* = 7.5 Hz, H-1), 4.32 (dd, 1H, *J* = 6.0,12.3 Hz), 3.90 (dd, 1H, *J* = 12.0, 2.0 Hz), 3.82 (s, 3H, -OCH_3_), 3.72 (dd, 1H, *J* = 5.1, 12.0 Hz), 3.45–3.24 (m, 4H). HRMS (ESI-TOF) calcd. for C_16_H_22_O_7_Na(M+Na)^+^ 349.1263; found 349.1259.

*3,5-Dimethoxycinnamyl β-D-glucopyranoside* (**10c**). Yield: 53%. [*α*]^25^_D_= –38.7 (*c* = 1.0, MeOH). ^1^H-NMR (300 MHz, methanol-*d_6_*): *δ* 6.60 (d, 1H, *J* = 16.5 Hz, Ar-H), 6.36 (m, 2H), 4.51 (dd, 1H, *J* = 5.6, 13.3 Hz, =CH-CH_2_), 4.36 (d, 1H, *J* = 7.7 Hz, =CH-CH_2_), 4.31 (dd, 1H, *J* = 6.7, 12.9 Hz, H-1), 3.88 (d, 1H, *J* = 11.6 Hz), 3.76 (s, 3H, -OCH_3 _× 2), 3.68 (dd, 1H, *J* = 5.2, 12.4 Hz, H-1), 3.38–3.21 (m, 4H). HRMS (ESI-TOF) calcd. for C_18_H_25_O_10 _(M+HCOO)^–^ 401.1448; found 401.1445.

*3,4-Dimethoxycinnamyl β-D-glucopyranoside* (**10d**). Yield: 73%. [*α*]^25^_D_= –38.2 (*c* = 1.0, MeOH). ^1^H-NMR (300 MHz, methanol-*d_6_*): *δ* 7.05 (d, 1H, *J* = 1.8 Hz, Ar-H), 6.95 (dd, 1H, *J* = 1.8, 8.4 Hz, Ar-H), 6.88 (d, 1H, *J* = 8.4 Hz, =CH), 6.60 (d, 1H, *J* = 15.9 Hz, =CH), 6.25 (dt, 1H, *J* = 6.3, 15.9 Hz, =CH), 4.51 (dd, 1H, *J* = 6.0, 12.9 Hz, =CH-CH_2_), 4.39 (d, 1H, *J* = 7.8 Hz, =CH-CH_2_), 4.33 (dd, 1H, *J* = 6.3, 12.6 Hz, H-1), 3.91 (d, 1H, *J* = 11.4 Hz), 3.84 (s, 3H, -OCH_3_), 3.81 (s, 3H, -OCH_3_), 3.71 (dd, 1H, *J* = 3.8, 11.7 Hz, H-1), 3.44–3.23 (m, 4H). HRMS (ESI-TOF) calcd. for C_17_H_24_O_8_Na(M+Na)^+^ 379.1369; found 379.1372.

*2,3-Dimethoxycinnamyl β-D-glucopyranoside* (**10e**). Yield: 53%. [*α*]^25^_D_= –34.8 (*c* = 1.0, MeOH). ^1^H-NMR (300 MHz, methanol-*d_6_*): *δ* 7.13 (dd, 1H, *J* = 7.8, 1.2 Hz, Ar-H), 7.03 (t, 1H, *J* = 8.1 Hz, Ar-H), 6.95 (d, 1H, *J* = 15.6 Hz, =CH), 6.91 (d, 1H, *J* = 7.5 Hz, =CH), 6.36 (dt, 2H, *J* =6.0, 16.2 Hz, =CH), 4.53 (dd, 1H, *J* = 5.1, 12 Hz, =CH-CH_2_), 4.39 (d, 1H, *J* = 7.5 Hz, =CH-CH_2_), 4.33 (d, 1H, *J* = 6.6 Hz, H-1), 3.90 (d, 1H, *J* = 11.7 Hz), 3.85 (s, 3H, -OCH_3_), 3.77 (s, 3H, -OCH_3_), 3.70 (dd, 1H, *J* = 5.1, 12 Hz, H-1), 3.43–3.23 (m, 4H). HRMS (ESI-TOF) calcd. for C_34_H_48_O_16_Na (2M+Na)^+^ 735.2840; found 735.2833.

*4-Trifluoromethycinnamyl β-D-glucopyranoside* (**10f**). Yield: 49%. [*α*]^25^_D_= –29.9 (*c* = 1.0, MeOH). ^1^H-NMR (300 MHz, methanol-*d_6_*): *δ* 7.60 (s, 4H, Ar-H × 4), 6.77 (d, 1H, *J* = 16.0 Hz, =CH), 6.52 (dt, 1H, *J* = 5.6, 16.0 Hz, =CH), 4.57 (dd, 1H, *J* = 13.4, 5.0 Hz, =CH-CH_2_), 4.39 (d, 1H, *J* = 7.5 Hz, H-1), 4.36 (dd, 1H, *J* = 5.3, 13.4 Hz, =CH-CH_2_), 3.91(d, 1H, *J* = 12.5 Hz), 3.70 (dd, 1H, *J* = 5.2, 11.8 Hz), 3.45–3.18 (m, 4H). HRMS (ESI-TOF) calcd. for C_17_H_20_O_8_F_3 _(M+HCOO)^–^ 409.1110; found 409.1104.

*3,4-Methylenedioxycinnamyl β-D-glucopyranoside* (**10g**). Yield: 51%. [*α*]^25^_D_= –36.8 (*c* = 1.0, MeOH). ^1^H-NMR (300 MHz, methanol-*d_6_*): *δ* 8.10 (d, *J* = 3.9 Hz, 1H), 7.47 (t, 1H, *J* = 3.9 Hz, =CH), 6.97 (d, 1H, *J* = 0.6 Hz), 6.83 (dd, 1H, *J* = 3.9, 0.6 Hz), 6.74 (d, 1H, *J* = 3.9 Hz), 6.58 (d, 1H, *J* = 8.1 Hz), 6.20 (dt, 1H, *J* = 3.0, 8.1 Hz), 5.92 (s, 2H, -CH_2_-), 4.90 (dd, 1H, *J* =0.3, 2.7 Hz, =CH-CH_2_), 4.37 (d, 1H, *J* = 3.9 Hz, H-1), 4.29 (dd, 1H, *J* =3.6, 6.3 Hz), 3.89 (d, 1H, *J* = 3.0 Hz), 3.69 (dd, 1H, *J* = 6.0, 2.7 Hz), 3.40–3.23 (m, 4H). HRMS (ESI-TOF) calcd. for C_16_H_20_O_8_Na(M+Na)^+^ 363.1056; found 363.1057.

*3,4,5-Trimethoxycinnamyl β-D-glucopyranoside* (**10h**). Yield: 57%. [*α*]^25^_D_= –38.2 (*c* = 1.0, MeOH). ^1^H-NMR (300 MHz, methanol-*d_6_*): *δ* 6.71 (s, 2H), 6.60 (d, 1H, *J* = 15.9 Hz, Ar-H), 6.30 (dt, 1H, *J* = 6.0, 9.7 Hz, =CH), 4.51 (dd, 1H, *J* = 5.0, 12.9 Hz, =CH-CH_2_), 4.37 (d, 1H, *J* = 7.7 Hz, =CH-CH_2_), 4.31 (dd, 1H, *J* = 6.5, 13.0 Hz, H-1), 3.88 (d, 1H, *J* = 11.8 Hz), 3.82 (s, 3H, -OCH_3 _× 2), 3.73 (s, 3H, -OCH_3_), 3.69 (dd, 1H, *J* = 5.0, 11.9 Hz, H-1), 3.40–3.22 (m, 4H). HRMS (ESI-TOF) calcd. for C_18_H_26_O_9_Na (M+Na)^+^ 409.1475; found 409.1455.

*2-Fluorocinnamyl β-D-glucopyranoside* (**10i**). Yield: 55%. [*α*]^25^_D_= –39.0 (*c* = 1.0, MeOH). ^1^H-NMR (300 MHz, methanol-*d_6_*): *δ* 7.47 (t, 1H, *J* = 7.8 Hz, Ar-H), 7.16 (q, 1H, *J* = 7.8 Hz, Ar-H), 7.06 (t, 1H, *J* = 7.5 Hz, =CH), 6.98 (t, 1H, *J* = 8.4 Hz, =CH), 6.76 (d, 1H, *J* = 16.2 Hz, =CH), 6.40 (dt, 1H, *J* = 6.0, 16.2 Hz, =CH-CH_2_), 4.50 (dd, 1H, *J* = 5.4, 12.9 Hz, =CH-CH_2_), 4.32 (d, 1H, *J* = 7.8 Hz, H-1), 4.28 (dd, 1H, *J* = 4.8, 11.4 Hz), 3.83 (d, 1H, *J* = 11.4 Hz), 3.63 (dd, 1H, *J* = 5.1, 12.0 Hz), 3.36–3.17 (m, 4H). HRMS (ESI-TOF) calcd. for C_16_H_20_O_8_F (M+HCOO)^–^ 359.1142; found 359.1145.

*4-Chlorocinnamyl* *β-D-glucopyranoside* **(10j**). Yield: 81%. [*α*]^25^_D_= –35.5 (*c* = 1.0, MeOH).^ 1^H-NMR (300 MHz, methanol-*d_6_*): *δ* 7.39 (d, 2H, *J* = 8.4 Hz, Ar-H × 2), 7.29 (d, 2H, *J* = 8.5 Hz, Ar-H × 2), 6.66 (d, 1H, *J* = 16.0 Hz, =CH), 6.38 (dt, 1H, *J* = 6.0, 16.0 Hz, =CH), 4.52 (dd, 1H, *J* = 5.3, 12.8 Hz, =CH-CH_2_), 4.36 (d, 1H, *J* = 7.7 Hz, H-1), 4.30 (dd, 1H, *J* = 5.3, 12.8 Hz, =CH-CH_2_), 3.88 (d, 1H, *J* = 12.5 Hz), 3.67 (dd, 1H, *J* = 11.8, 5.2 Hz), 3.35–3.18 (m, 4H). HRMS (ESI-TOF) calcd. for C_16_H_20_O_8_Cl(M+HCOO)^–^ 375.0847; found 375.0857.

*3,4-Difluorocinnamyl β-D-glucopyranoside* (**10k**). Yield: 49%. [*α*]^25^_D_= –40.0 (*c* = 1.0, MeOH). ^1^H-NMR (300 MHz, methanol-*d_6_*): *δ* 7.28 (dd, 1H, *J* = 7.3, 10.6 Hz, Ar-H), 7.14-7.09 (m, 2H), 6.58 (d, 1H, *J* = 16.0 Hz, =CH), 6.28 (dt, 1H, *J* = 5.9, 16.0 Hz, =CH), 4.45 (dd, 1H, *J* = 5.3, 13.2 Hz, =CH-CH_2_), 4.28 (d, 1H, *J* = 7.7 Hz, H-1), 4.25 (dd, 1H, *J* = 6.2, 14.7 Hz), 3.81 (d, 1H, *J* = 11.5 Hz), 3.61 (dd, 1H, *J* = 5.2, 11.9 Hz), 3.30–3.14 (m, 4H). HRMS (ESI-TOF) calcd. for C_16_H_19_O_8_F_2 _(M+HCOO)^–^ 377.1048; found 377.1035.

*4-Bromocinnamyl β-D-glucopyranoside* (**10l**). Yield: 70%. [*α*]^25^_D_= –31.2 (*c* = 1.0, MeOH). ^1^H-NMR (300 MHz, methanol-*d_6_*): *δ* 7.45(d, 2H, *J* = 8.4 Hz, Ar-H), 7.34 (d, 2H, *J* = 8.4 Hz, Ar-H), 6.67 (d, 1H, *J* = 16.0 Hz, =CH), 6.41 (dt, 1H, *J* = 5.8, 15.9 Hz, =CH), 4.53 (dd, 1H, *J* = 5.2, 13.2 Hz, =CH-CH_2_), 4.37 (d, 1H, *J* = 7.8 Hz, =CH-CH_2_), 4.33 (dd, 1H, *J* = 6.8, 13.8 Hz, H-1), 3.90 (d, 1H, *J* = 11.5 Hz), 3.69 (dd, 1H, *J* = 4.8, 11.9 Hz, H-1), 3.39–3.22 (m, 4H). HRMS (ESI-TOF) calcd. for C_16_H_20_O_8_Br(M+HCOO)^–^ 479.0342; found 479.0340.

*Cinnamyl β-D-glucopyranoside* (**10m**). Yield: 61%. [*α*]^25^_D_= –24.2 (*c* = 1.0, MeOH). ^1^H-NMR (300 MHz, DMSO-*d_6_*): *δ* 7.85 (d, 2H, *J* = 7.2 Hz, Ar-H), 7.74 (t, 2H, *J* = 7.1 Hz, Ar-H), 7.66 (d, 1H, *J* = 7.2 Hz, =CH), 7.18 (d, 1H, *J* = 16.0 Hz, =CH), 5.50 (d, 1H, *J* = 4.9 Hz, =CH), 5.35 (dd, 2H, *J* = 4.4, 12.8 Hz, =CH-CH_2_), 4.95 (t, 1H, *J* = 5.8 Hz, H-1), 4.83 (dd, 1H, *J* = 6.6, 12.3 Hz, =CH-CH_2_), 4.63 (d, 2H, *J* = 7.7 Hz), 3.87 (m, 1H), 3.48 (m, 4H). (ESI-TOF) calcd. for C_15_H_20_O_6_Na (M+Na)^+^ 319.1158; found 319.1158.

*3-Bromo-4-ethoxy-5-methoxycinnamylcinnamyl β-D-glucopyranoside* (**10n**). Yield: 43%. [*α*]^25^_D_= –31.2 (*c* = 1.0, MeOH). ^1^H-NMR (300 MHz, methanol-*d_6_*): *δ* 7.18 (d, 1H, *J* = 1.7 Hz, Ar-H), 7.03 (d, 1H, *J* = 1.7 Hz, Ar-H), 6.59 (d, 1H, *J* = 15.9 Hz, =CH), 6.32 (dt, 1H, *J* = 6.0, 15.9 Hz, =CH-CH_2_), 4.54 (dd, 1H, *J* = 5.5, 13.0 Hz, =CH-CH_2_), 4.36 (d, 1H, *J* = 7.7 Hz, H-1), 4.31 (dd, 1H, *J* = 6.5, 13.3 Hz), 4.02 (q, 2H, *J* = 7.1 Hz), 3.86 (s, 3H, -OCH_3_),3.67 (dd, 1H, *J* = 5.2, 11.9 Hz, =CH-CH_2_), 3.40-3.20 (m, 4H), 1.35 (t, 3H, *J* = 7.2 Hz, -CH_3_). HRMS (ESI-TOF) calcd. for C_19_H_26_O_10_Br(M+HCOO)^– ^493.0709; found 493.0700.

*4-Hydroxycinnamyl β-D-glucopyranoside* (**16a**). Yield: 46%. [*α*]^25^_D_= –32.6 (*c* = 1.0, MeOH). ^1^H-NMR (300 MHz, methanol-*d_6_*): *δ* 7.23 (d, 2H, *J* = 8.6 Hz, Ar-H), 6.72 (d, 2H, *J* = 8.5 Hz, Ar-H), 6.55 (d, 1H, *J* = 15.9 Hz, =CH), 6.15 (dt, 1H, *J* = 5.8, 15.9 Hz**),** 4.47 (dd, 1H, *J* = 6.0, 12.8 Hz, =CH-CH_2_), 4.36 (d, 1H, *J* = 7.7 Hz, H-1), 4.26 (dd, 1H, *J* = 6.8, 12.4 Hz), 3.89 (d, 1H, *J* = 12.9 Hz), 3.69 (dd, 1H, *J* = 5.2, 12.0 Hz), 3.39–3.21 (m, 4H). HRMS (ESI-TOF) calcd. for C_15_H_20_O_7 _Na (M+Na)^+^ 335.1107; found 335.1088.

*3-Hydroxycinnamyl β-D-glucopyranoside* (**16b**). Yield: 51%. [*α*]^25^_D_= –35.2 (*c* = 1.0, MeOH). ^1^H-NMR (300 MHz, methanol-*d_6_*): *δ* 7.12 (t, 1H, *J* = 7.8 Hz, Ar-H), 6.88 (d, 2H, *J* = 9.2 Hz, Ar-H), 6.69 (dd, 1H, *J* = 2.1, 8.4 Hz, =CH), 6.61 (d, 1H, *J* = 15.9 Hz, =CH), 6.32 (dt, 1H, *J* = 6.3, 15.9 Hz, =CH), 4.51 (dd, 1H, *J* = 5.7, 12.6 Hz, =CH-CH_2_), 4.39 (d, 1H, *J* = 7.8 Hz, =CH-CH_2_), 4.31 (dd, 1H, *J* = 6.6, 13.5 Hz, H-1), 3.95 (d, 1H, *J* = 12.0 Hz), 3.71 (dd, 1H, *J* = 5.1, 12 Hz, H-1), 3.41-3.23 (m, 4H). HRMS (ESI-TOF) calcd. for C_16_H_21_O_9 _(M+HCOO)^–^ 357.1186; found 357.1173.

*4-Hydroxy-3-methoxycinnamyl β-D-glucopyranoside* (**16c**). Yield: 51%. [*α*]^25^_D_= –36.2 (*c* = 1.0, MeOH). ^1^H-NMR (300 MHz, methanol-*d_6_*): *δ* 7.01 (d, 1H, *J* = 1.5 Hz, Ar-H), 6.85 (dd, 1H, *J* = 1.5, 8.1 Hz, Ar-H), 6.72 (d, 1H, *J* = 8.1 Hz, =CH), 6.57 (d, 1H, *J* = 15.9 Hz, =CH), 6.18 (dt, 1H, *J* = 6.3, 15.9 Hz, =CH), 4.49 (dd, 1H, *J* = 6.0, 13.3 Hz, =CH-CH_2_), 4.37 (d, 1H, *J* = 7.5 Hz, H-1), 4.29 (dd, 1H, *J* = 6.6, 12.3 Hz, =CH-CH_2_), 3.91 (d, 1H, *J* = 10.8 Hz), 3.86 (s, 3H, -OCH_3_), 3.68 (dd, 1H, *J* = 5.1, 11.7 Hz, =CH-CH_2_), 3.40-3.19 (m, 4H). HRMS (ESI-TOF) calcd. for C_17_H_23_O_10 _(M+HCOO)^– ^387.1291; found 387.1284.

*3-Hydroxy-4-methoxycinnamyl β-D-glucopyranoside* (**16d**). Yield: 61%. [*α*]^25^_D_= –35.7 (*c* = 1.0, MeOH). ^1^H-NMR (300 MHz, methanol-*d_6_*): *δ* 6.96 (d, 1H, *J* = 1.5 Hz, Ar-H), 6.87 (dd, 1H, *J* = 1.5, 8.0 Hz, Ar-H), 6.73 (d, 1H, *J* = 8.1 Hz, =CH), 6.56 (d, 1H, *J* = 15.9 Hz, =CH), 6.18 (dt, 1H, *J* = 6.3, 15.9 Hz, =CH), 4.44 (dd, 1H, *J* = 6.0, 13.3 Hz, =CH-CH_2_), 4.33 (d, 1H, *J* = 7.5Hz, H-1), 4.29 (dd, 1H, *J* = 6.6, 12.3 Hz, =CH-CH_2_), 3.91 (d, 1H, *J* = 10.8 Hz), 3.86 (s, 3H, -OCH_3_), 3.66 (dd, 1H, *J* = 5.1, 11.7 Hz, =CH-CH_2_), 3.40-3.20 (m, 4H). HRMS (ESI-TOF) calcd. for C_17_H_23_O_10 _(M+HCOO)^– ^387.1291; found 387.1283.

*Cinnamyl 6-trityl-O-β-D-glucopyranoside* (**17a**). A solution of **10m** (1.25 g, 3.8 mmol), trityl chloride (2.24 g, 8.1 mmol), triethylamine (0.98 g, 9.8 mmol), DMAP (0.31 g, 2.5 mmol) and powered 4 Å molecular sieves (2.5 g) in DMF (12 mL) was stirred overnight at room temperature under nitrogen. After another 12 h stirring, the yellow cloudy solution was poured into ice-water and extracted with CH_2_Cl_2_. The organic extracts were washed with saturated ammonium chloride solution and water, dried (Na_2_SO_4_). After removal of the solvents, the yellowish solid was subjected to column chromatography on silica gel with petroleum-ethyl acetate (3:1) and ethyl acetate as eluents. Concentrating the ethyl acetate part gave the product as a white amorphous solid. Yield: 1.14 g (56%). m.p. 68–70 °C; [*α*]^25^_D_= –48.8 (*c* 1.0, MeOH); ^1^H-NMR (600 MHz, DMSO-*d_6_*): *δ* 7.50–7.20 (m, 20H, Ar-H × 20), 6.70 (d, 1H, *J* = 16.2 Hz, =CH), 6.44 (dt, 1H, *J* = 16.2, 6.0 Hz, =CH), 5.15 (d, 1H, *J* = 4.8 Hz), 4.98 (d, 1H, *J* = 4.8 Hz), 4.86 (d, 1H, *J* = 5.4 Hz,), 4.52 (dd, 1H, *J* = 5.4, 13.2 Hz, =CH-CH_2_), 4.35 (dd, 1H, *J* = 5.4, 13.2 Hz, =CH-CH_2_), 4.34 (d, 1H, *J* = 7.8 Hz), 3.28 (d, 1H, *J* = 9.6 Hz), 3.15 (m, 1H), 3.07 (m, 3H). ^13^C-NMR (75 MHz, DMSO-*d_6_*): *δ* 144.1 (3C, 3Ar-C), 136.5 (Ar-C), 131.6 (=CH)), 128.8 (4C, 4Ar-C), 128.5 (4C, 4Ar-C ), 128.0 (4C, 4Ar-C), 127.8 ( =CH), 127.1 (4C, 4Ar-C), 126.5 (4C, 4Ar-C), 102.2 (C-1), 85.7 (Ph_3_C), 77.0 (C-3), 75.3 (C-5), 73.6 (C-2), 70.4 (C-4), 68.6 (=C-CH_2_), 63.8 (C-6). HRMS (ESI-TOF): calcd. for C_35_H_35_O_8 _(M+HCOO)^–^ 583.2332; found 583.2332.

*4-Methoxycinnamyl 6-trityl-O-β-D-glucopyranoside* (**17b**).Prepared according to synthetic method of 1**7a** from **10a** (1.23 g, 3.8 mmol). Yield: 1.15 g (64%).m.p. 58–60 °C; [*α*]^25^_D_= –25.4( *c* 1.0, MeOH); ^1^H-NMR (600 MHz, DMSO-*d_6_*): *δ* 7.90 (d, 2H, *J* = 7.8 Hz, Ar-H × 2), 7.73 (d, 2H, *J* = 7.2 Hz, Ar-H × 2), 7.66 (d, 2H, *J* = 7.8 Hz, Ar-H × 2), 7.58 (m, 2H, Ar-H × 2), 7.49 (t, 1H, *J* = 7.2 Hz, Ar-H), 7.43 (t, 2H, *J* = 7.8 Hz, Ar-H × 2), 7.40–7.35 (m, 11H, Ar-H × 11), 7.20–7.15 (m, 8H, Ar-H × 8), 7.15–7.10 (m, 4H, Ar-H × 4), 6.81 (d, 2H, *J* = 9.0 Hz, Ar-H × 2), 6.51 (d, 1H, *J* = 16.2 Hz, =CH), 6.16 (dt, 1H, *J* = 16.2, 5.4 Hz, =CH), 5.90 (t, 1H, *J* = 5.4 Hz), 7.25 (m, 6H, Ar-H × 6), 7.25–7.21 (m, 3H, Ar-H × 3), 6.85 (d, 2H, *J* = 8.4 Hz, Ar-H × 2), 6.62 (d, 1H, *J* = 16.2 Hz, =CH), 6.28 (dt, 1H, *J* = 16.2, 6.0 Hz, =CH), 5.13 (d, 1H, *J* = 4.8 Hz), 4.98 (d, 1H, *J* = 4.8 Hz), 4.85 (d, 1H, *J* = 5.4 Hz), 4.49 (dd, 1H, *J* = 5.4, 13.2 Hz, =CH-CH_2_), 4.33 (d, 1H, *J* = 7.8 Hz), 4.32 (dd, 1H, *J* = 5.4, 13.2 Hz, =CH-CH_2_), 3.73 (s, 3H, -OCH_3_), 3.27 (d, 1H, *J* = 9.6 Hz), 3.15 (m, 1H), 3.07 (m, 3H). ^13^C-NMR (75 MHz, DMSO-*d_6_*): *δ* 159.1 (Ar-C), 144.1 (3C, 3Ar-C),131.6 (Ar-C), 129.4 (=CH), 129.2 (=CH), 128.5 (6C, 6Ar-C), 127.9 (6C, 6Ar-C), 127.8 (2C, 2Ar-C), 127.1 (3C, 3Ar-C), 114.2 (2C, 2Ar-C ), 102.1 (C-1), 85.7 (Ph_3_C), 77.0 (C-3), 75.3 (C-5), 73.7 (C-2), 70.5 (C-4), 68.8 (=C-CH_2_), 63.9 (C-6), 55.2 (OCH_3_). HRMS (ESI-TOF): calcd. for C_36_H_37_O_9_ (M+HCOO)^– ^613.2438; found 613.2433.

*Cinnamyl 6-trityl-2,3,4-tri-O-benzoyl-β-D-glucopyranoside* (**18a**). Cinnamyl 6-trityl-*O*-β-D-gluco-pyranoside (4.4 g, 8.16 mmol) in pyridine (50 mL) was cooled to 0 °C, and then benzoyl chloride (5.71 g, 40.8 mmol) was added dropwise over 30 min. The reaction temperature was raised to room temperature. After 24 h water (50 mL) was added to the reaction mixture and stirring was continued for 30 min. The aq. solution was extracted with CH_2_Cl_2 _(3 × 50 mL) and the extracts were washed with saturated NaHCO_3_. The solution was evaporated under vacuum to give a yellow sticky solid which was dissolved in toluene and dehydrate by repeated azeotropic distillation to give the crude product (6.7 g). The product was purified using a short silica gel column eluted with petroleum-ethyl acetate (10:1), to give the title compound as a yellow oil (6.0 g, yield 87%). [*α*]^25^_D_+4.9 ( *c* 1.0, CDCl_3_); ^1^H-NMR (600 MHz, DMSO-*d_6_*): *δ* 7.99–7.14 (m, 35H, Ar-H × 35), 6.59 (d, 1H, *J* = 16.2 Hz, =CH), 6.35 (dt, 1H, *J* = 16.2, 5.4 Hz, =CH), 5.93 (t, 1H, *J* = 9.6 Hz), 5.74 (t, 1H, *J* = 9.6 Hz), 5.49 (d, 1H, *J* = 9.0 Hz), 5.27 (d, 1H, *J* = 7.8 Hz), 4.56 (dd, 1H, *J* = 5.4, 13.2 Hz, =CH-CH_2_), 4.42 (dd, 1H, *J* = 6.0, 13.8 Hz, =CH-CH_2_) 4.29 (d, 1H, *J* = 10.2 Hz), 3.35 (d, 1H, *J* = 10.2 Hz), 3.06 (dd, 1H, *J* = 3.0, 10.2 Hz).^13^C-NMR (75 MHz, DMSO-*d_6_*): *δ* 165.3 (PhCO-), 164.9 (PhCO), 164.5 (PhCO-), 143.5 (3C, 3Ar-C), 136.2 (Ar-C), 133.8 (3C, 3Ar-C), 132.9 (2Ar-C), 131.8 (=CH), 130.9 (Ar-C), 129.4 (3C, 3Ar-C), 129.2 (3C, 3Ar-C), 128.9 (3C, 3Ar-C), 128.7 (3C, 3Ar-C), 128.6 (6C, 6Ar-C), 128.2 (6C, 6Ar-C), 127.9 (4C, =CH, 3Ar-C), 127.0 (2C, 2Ar-C), 126.4 (2C, 2Ar-C), 125.5 (Ar-C), 99.2 (C-1), 85.9 (Ph_3_C), 73.6 (C-3), 72.3 (2C, C-5,2), 69.0 (2C, C-4, =C-CH_2_), 61.7 (C-6). HRMS (ESI-TOF): calcd. for C_56_H_47_O_11_Na (M+Na)^+^ 873.3040; found 873.3020.

*4-Methoxycinnamyl 6-trityl-2,3,4-tri-O-benzoyl-β-D-glucopyranoside* (**18b**). Prepared according to the synthetic method used for the preparation of **18a** from **17b** (5.7 g, 10 mmol). Yield: 7.93 g (90%). m.p. 88–90 °C; [*α*]^25^_D_+21.5 ( *c* 1.0, CDCl_3_); ^1^H-NMR (600 MHz, DMSO-*d_6_*): *δ* 7.90–7.10 (m, 32H, Ar-H × 32), 6.82 (d, 2H, *J* = 9.0 Hz, Ar-H × 2), 6.51 (d, 1H, *J* = 16.2 Hz, =CH), 6.17 (dt, 1H, *J* = 16.2, 5.4 Hz, =CH), 5.90 (t, 1H, *J* = 9.6 Hz), 5.70 (t, 1H, *J* = 9.6 Hz), 5.47 (t, 1H, *J* = 9.0 Hz), 5.24 (d, 1H, *J* = 7.8 Hz, H-1), 4.51 (dd, 1H, *J* = 5.4, 13.2 Hz, =CH-CH_2_), 4.42 (dd, 1H, *J* = 6.0, 13.2 Hz, =CH-CH_2_), 4.26 (dt, 1H, *J* = 2.4, 9.6 Hz), 3.33 (d, 1H, *J* = 9.0 Hz), 3.03 (dd, 1H, *J* = 3.6, 10.2 Hz).^13^C-NMR (75 MHz, DMSO-*d_6_*): *δ* 165.3 (PhCO-), 164.9 (PhCO-), 164.5 (PhCO-), 159.0 (2C, 2Ar-C), 143.5 (3C, 3Ar-C), 133.7 (3C, 3Ar-C), 131.8 (3C, 3Ar-C), 129.3 (2C, 2Ar-C), 129.2 (3C, 3Ar-C), 128.9 (3C, 3Ar-C), 128.8 (3C, 3Ar-C), 128.6 (=CH), 128.2 (6C, 6Ar-C), 127.9 (3C, 3Ar-C), 127.7 (3C, 3Ar-C), 127.0 (3C, 3Ar-C), 122.9 (=CH), 114.0 (2C, 2Ar-C), 99.2 (C-1), 86.0 (Ph_3_C), 73.7 (C-3), 72.3 (2C, C-5, 2), 69.1 (2C, C-4, =C-CH_2_), 61.7 (C-6), 55.1 (OCH_3_). HRMS (ESI-TOF): calcd. for C_56_H_49_O_10 _(M+H)^+^ 881.3320; found 881.3347.

*Cinnamyl 2,3,4-tri-O-benzoyl-β-D-glucopyranoside* (**19a**). Cinnamyl 6-trityl-2,3,4-tri-*O*-benzoyl-β-D-glucopyranoside (**18b**, 5.0 g, 5.9 mol) was dissolved in CH_2_Cl_2_ (30 mL) followed by the addition of 90% aq TFA (3.0 mL) and the solution was allowed to stir for 30 min at room temperature. Then the solution was poured into a separating funnel and washed successively with water (50 mL), saturated NaHCO_3_ (2 × 50 mL) and brine (50 mL). The organic layer was collected, dried (Na_2_SO_4_) and filtered. The filtrate was evaporated and the resulting crude material was purified by silica gel chromatography using petroleum-ethyl acetate (3:1) as eluent, affording pure compound (3.0 g, 84%) as a light yellow solid.m.p. 108–110 °C; [*α*]^25^_D_+2.9 (*c* 1.0, CDCl_3_);^1^H-NMR (600 MHz, DMSO-*d_6_*): *δ* 7.87–7.15 (m, 20H, Ar-H × 20), 6.51 (d, 1H, *J* = 16.2 Hz, =CH), 6.23 (dt, 1H, *J* = 5.4, 16.2 Hz, =CH), 5.93 (t, 1H, *J* = 9.6 Hz), 5.49 (d, 1H, *J* = 9.0 Hz), 5.36 (d, 1H, *J* = 9.0 Hz), 5.18 (d, 1H, *J* = 7.8 Hz), 5.06 (s, 1H, OH), 4.48 (dd, 1H, *J* = 5.4, 13.2 Hz, =CH-CH_2_), 4.32 (dd, 1H, *J* = 6.0, 13.8 Hz, =CH-CH_2_), 4.09 (m, 1H), 3.66 (m, 2H), 3.06 (dd, 1H, *J* = 3.0, 10.2 Hz). ^13^C-NMR (75 MHz, DMSO-*d_6_*): *δ* 165.4 (PhCO-), 165.0 (2C, 2PhCO-), 136.3 (Ar-C), 133.9 (3C, 3Ar-C), 131.8 (=CH), 129.4 (3C, 3Ar-C), 129.2 (2C, 2Ar-C), 129.0 (3C, 3Ar-C), 128.9 (2C, 2Ar-C), 128.7 (3C, 3Ar-C), 127.9 (=CH), 126.4 (3C, 3Ar-C), 125.5 (Ar-C), 99.3 (C-1), 74.2 (C-3), 73.9 (C-5), 72.3 (C-2), 69.6 (C-4), 69.2 (=C-CH_2_), 60.4 (C-6). HRMS (ESI-TOF): calcd. for C_37_H_33_O_11 _(M+HCOO)^–^ 653.2023; found 653.2028.

*4-Methoxycinnamyl 2,3,4-tri-O-benzoyl-β-D-glucopyranoside* (**19b**). Prepared according to synthetic method described for preparation of **19a** from **18b** (6.0 g, 6.8 mmol). Yield: 3.73 g (86%). m.p. 125–127 °C; [*α*]^25^_D_+6.8 (*c* 1.0, CDCl_3_); ^1^H-NMR (600 MHz, CDCl_3_): *δ* 8.10-7.10 (m, 17H, Ar-H × 17), 6.80 (d, 2H, *J* = 9.0 Hz, Ar-H × 2), 6.49 (d, 1H, *J* = 15.6 Hz, =CH), 6.00 (dt, 1H, *J* = 6.0, 15.0 Hz, =CH), 5.93 (t, 1H, *J* = 9.6 Hz), 5.55 (t, 1H, *J* = 9.0 Hz), 5.51 (t, 1H, *J* = 9.0 Hz), 4.94 (d, 1H, *J* = 7.8 Hz, H-1), 4.51 (dd, 1H, *J* = 5.4, 13.2 Hz, =CH-CH_2_), 4.34 (dd, 1H, *J* = 6.0, 13.8 Hz, =CH-CH_2_), 3.87 (m, 1H), 3.79 (s, 3H, OCH_3_), 3.82–3.78 (m, 2H), 3.48 (s, 1H, -OH). ^13^C-NMR (75 MHz, CDCl_3_): *δ* 166.0 (PhCO-), 165.8 (PhCO-), 165.1 (PhCO-), 159.3 (Ar-C), 133.6 (Ar-C), 133.2 (Ar-C), 132.8 (Ar-C), 130.1 (2C, 2Ar-C), 129.8 (2C, 2Ar-C), 129.7 (2C, 2Ar-C), 129.6 (2C, 2Ar-C), 129.2 (Ar-C), 128.8 (3C, 3Ar-C), 128.2 (2C, 2Ar-C), 127.8 (2C, 2Ar-C), 127.2 (2C, 2Ar-C), 126.9 (Ar-C), 122.1 (Ar-C), 113.9 (2C, 2Ar-C), 99.6 (C-1), 74.6 (C-3), 72.7 (C-5), 71.8 (C-2), 70.1 (C-4), 69.5 (=C-CH_2_), 61.3 (C-6), 55.2 (OCH_3_). HRMS (ESI-TOF): calcd. for C_38_H_35_O_12 _(M+HCOO)^–^ 638.2129; found 638.2104.

*Rosavin* (**20**). A suspension of cinnamyl 2,3,4-tri-*O*-benzoyl-β-D-glucopyranoside (100 mg, 0.164 mmol), 2,3,4-tri-*O*-benzoyl-α-L-arabinopyranosyl tricholoroacetimidate (119 mg, 0.197 mmol) and powdered 4 Å molecular sieves (1.0 g) in dry CH_2_Cl_2_ (20 mL) was stirred for 30 min at −20 °C. A dry CH_2_Cl_2_ solution (0.2 mL) containing TMSOTf (1.8 µL, 0.01 mmol) was added dropwise. The mixture was stirred for 1 h before Et_3_N (0.1 mL) was added to quench the reaction, and then the mixture was diluted with CH_2_Cl_2_ (20 mL) and passed through a sintered-glass funnel. The resulting solution was concentrated and the resulting residue was dissolved in dry CH_2_Cl_2_- MeOH (1:2, 30 mL). NaOMe (108 mg, 2.0 mmol) was added. The solution was stirred at room temperature for 2 h and then neutralized with Dowex H^+ ^resin to pH 7. The resin was filtered and the filtrate was concentrated. The residue was subjected to a silica gel PTLC to give the product as a white powder. Yield: 69 mg (79%).m.p. 170–173 °C; [*α*]^25^_D_-54.2 [*c* 0.7, CHCl_3_:MeOH (1:1)]. Lit. [19] [*α*]^20^_D_-56.5 [*c* 0.7, CHCl_3_:MeOH (1:1)]; ^1^H-NMR (600 MHz, MeOH-*d_4_*): *δ* 7.36 (d, 2H, *J* = 7.2 Hz, Ar-H × 2), 7.26 (t, 2H, *J* = 7.2 Hz, Ar-H × 2), 7.15 (t, 1H, *J* = 7.2 Hz, Ar-H), 6.61 (d, 1H, *J* = 15.6 Hz, =CH), 6.31 (dt, 1H, *J* = 6.0, 16.2 Hz, =CH), 4.45 (dd, 1H, *J* = 6.0, 12.6 Hz, =CH-CH_2_), 4.37 (d, 1H, *J* = 7.8 Hz, H-1′), 4.36 (d, 1H, *J* = 6.8 Hz, H-1′′), 4.35 (dd, 1H, *J* = 6.0, 12.6 Hz, =CH-CH_2_), 4.11 (d, 1H, *J* = 10.9 Hz), 3.94 (dd, 1H, *J* = 1.8 Hz, 11.4 Hz), 3.77 (d, 1H, *J* = 2.0, 3.0 Hz), 3.75 (1H, dd, *J* = 6.0, 11.4 Hz), 3.61 (t, 1H, *J* = 7.2 Hz), 3.55 (m, 2H), 3.35 (m, 1H), 3.33 (m, 1H), 3.25 (m, 1H), 3.20 (t, 1H, *J* = 7.0 Hz).^ 13^C-NMR (150 MHz, MeOH-*d_4_*): *δ* 138.3 (Ar-C), 133.8 ( =CH), 129.7 (2C, 2Ar-C), 128.7 (Ar-C), 127.6 (2C, 2Ar-H), 126.7 (=CH), 105.2 (C-1′′), 103.4 (C-1′), 78.1 (C-3′), 76.9 (C-5′), 75.1 (C-2′), 74.3 (C-3′′), 72.5 (C-2′′), 71.8 (C-4′), 70.9 (-CH_2_-), 69.6 (C-4′′, C-6′), 66.7 (C-5′′). HRMS (ESI-TOF): calcd. for C_21_H_29_O_12 _(M+HCOO)^–^ 473.1659; found 473.1656.

*Cinnamyl 6-O-(β-D-xylopyranosyl)-β-D-glucopyranoside* (**21**). Prepared according to the synthetic method described for the preparation of **20** from **19a** (100 mg, 0.164 mmol). Yield: 68 mg (78%). m.p. 173–175 °C; [*α*]^25^_D_−67.9 ( *c* 1.0, MeOH). ^1^H-NMR (600 MHz, MeOH-*d_4_*): *δ* 7.33 (d, 2H, *J* = 7.8 Hz, Ar-H × 2), 7.29 (t, 2H, *J* = 7.8 Hz, Ar-H × 2), 7.20 (t, 1H, *J* = 7.2 Hz, Ar-H), 6.68 (d, 1H, *J* = 15.6 Hz, =CH), 6.36 (dt, 1H, *J* = 6.0, 16.2 Hz, =CH), 4.51 (dd, 1H, *J* = 6.0, 12.6 Hz, =CH-CH_2_), 4.38 (d, 1H, *J* = 7.8 Hz, H-1′), 4.36 (d, 1H, *J* = 7.2 Hz, H-1′′), 4.35 (dd, 1H, *J* = 6.0, 12.6 Hz, =CH-CH_2_), 4.11 (d, 1H, *J* = 1.8, 9.6 Hz), 3.86 (dd, 1H, *J* = 5.6, 11.4 Hz), 3.77 (dd, 1H, *J* = 6.0, 11.4 Hz), 3.55–3.45 (m, 1H), 3.40–3.32 (m, 3H), 3.28–3.13 (m, 3H).^13^C-NMR (75 MHz, MeOH-*d_4_*): *δ* 138.1 (Ar-C), 133.9 (=CH), 129.5 (2C, 2Ar-C), 128.7 (Ar-C), 127.5 (2C, 2Ar-H), 126.7 (=CH), 105.4 (C-1′′), 103.3 (C-1′), 77.8 (C-3′′), 77.5 (C-3′), 76.8 (C-5′), 74.9 (C-2′), 74.6 (C-2′′), 71.4 (C-4′), 71.1 (C-4′′), 70.9 (-CH_2_-), 69.8 (C-6′), 66.8 (C-5′′). HRMS (ESI-TOF): calcd. for C_21_H_29_O_12 _(M+HCOO)^–^ 473.1659; found 473.1656.

*4-Methoxycinnamyl 6-O-(α-L-arabinopyranosyl)-β-D-glucopyranoside* (**22**). Prepared according to synthetic method described for the preparation of **20** from **19b** (100 mg, 0.156 mmol). Yield: 71 mg (81%). m.p. 93–95 °C; [*α*]^25^_D_−40.2 (*c* 1.0, MeOH). ^1^H-NMR (600 MHz, MeOH-*d_4_*,): *δ* 7.37 (d, 2H, *J* = 8.3 Hz), 6.83 (d, 2H, *J* = 8.3 Hz), 6.60 (d, 1H, *J* = 15.9 Hz), 6.20 (td, 1H, *J* = 6.8, 15.9 Hz), 4.48 (dd, 1H, *J* = 5.8, 12.6 Hz), 4.36 (d, 1H, *J* = 7.8 Hz, H-1′), 4.33 (d, 1H, *J* = 6.8 Hz, H-1′′), 4.30 (dd, 1H, *J* = 7.8, 12.6 Hz), 3.88 (dd, 1H, *J* = 3.0, 12.4 Hz), 3.82-3.78 (m, 1H), 3.78 (s, 3H), 3.74 (dd, 1H, *J* = 5.8, 11.4 Hz), 3.63 (t, 1H, *J* = 6.8 Hz), 3.55–3.51 (m, 2H), 3.46–3.40 (m, 1H), 3.37–3.33 (m, 3H), 3.25–3.20 (m, 1H); ^13^C-NMR (75 MHz, MeOH-*d_4_*): *δ* 160.9 (Ar-C), 133.7 (=CH), 130.9 (Ar-C), 128.8 (2C, 2Ar-C), 124.3 (=CH), 115.0 (2C, 2Ar-C), 105.1 (C-1′′), 103.2 (C-1′), 78.0 (C-3′), 76.9 (C-5′), 75.1 (C-2′), 74.2 (C-3′′), 72.4 (C-2′′), 71.7 (C-4′), 71.1 (-OCH_2_-), 69.5 (C-6′), 69.4 (C-4′′), 66.7 (C-5′′), 55.7 (OCH_3_); HRMS (ESI-TOF): C_22_H_31_O_13 _(M+HCOO)^– ^503.1765; found 503.1773.

*Cinnamyl 6-O-(α-L-rhamnopyranosyl)-β-D-glucopyranoside* **(23**). Prepared according to synthetic method described for the preparation of **20** from **19a** (100 mg, 0.164 mmol). Yield: 74 mg (83%). m.p. 111–112 °C; [*α*]^25^_D_−59.2 (*c* 1.0, MeOH);^ 1^H-NMR (600 MHz, MeOH-*d_4_*,): *δ* 7.35 (d, 2H, *J* = 7.2 Hz, Ar-H × 2), 7.25 (t, 2H, *J* = 7.2 Hz, Ar-H × 2), 7.17 (t, 1H, *J* = 7.2 Hz, Ar-H), 6.62 (d, 1H, *J* = 15.6 Hz, =CH), 6.31 (dt, 1H, *J* = 6.0, 16.2 Hz, =CH), 4.45 (dd, 1H, *J* = 6.0, 12.6 Hz, =CH-CH_2_), 4.74 (d, 1H, *J* = 1.2 Hz, H-1′′), 4.32 (d, 1H, *J* = 7.8 Hz, H-1′), 4.35 (dd,1H, *J* = 6.0, 12.6 Hz, =CH-CH_2_), 3.94 (dd, 1H, *J* = 1.8 Hz, 11.4 Hz, H-6′), 3.86 (t, 1H, *J* = 1.8 Hz, H-2′′), 3.77 (d, 1H, *J* = 2.0, 3.0 Hz, H-5′′), 3.67 (m, 1H, H-3′′), 3.60 (1H, dd *J* = 6.0, 11.4 Hz, H-6′), 3.39 (m, 1H, H-5′), 3.37 (m, 1H, H-4′), 3.35 (m, 1H, H-4′′), 3.33 (m, 1H, H-6′), 3.25 (1H, m, H-3′), 3.20 (t, 1H, *J* = 9.0 Hz, H-2′), 1.24 (d, 3H, *J* = 7.8 Hz, CH_3_). ^13^C-NMR (75 MHz, MeOH-*d_4_*): *δ* 138.0 (Ar-C), 133.9 (=CH), 129.5 (2C, 2Ar-C), 128.7 (Ar-C), 127.5 (2C, 2Ar-H), 126.4 (=CH), 103.1 (C-1′), 102.2 (C-1′′), 77.9 (C-5′), 76.7 (C-4′), 74.9 (C-2′), 73.9 (C-4′′), 72.2 (C-3′′), 72.1 (C-2′′), 71.5 (C-3′), 70.7 (-CH_2_-), 69.7 (C-5′′), 68.1 (C-6′), 18.0 (-CH_3_). HRMS (ESI-TOF): calcd. for C_22_H_31_O_12_ (M+HCOO)^– ^487.1816; found 487.1828.

*Assay of the in vitro AChE/XOD inhibitory activity*: The tests of *in vitro* inhibitory activity of these PPGs against AChE (from drosophila, Jing Peng Bio-Pesticide Co., Ltd. Shandong, P. R. China) and Xanthine Oxidase (from buttermilk, Sigma Co., Ltd. X4875) were carried out by using AChE (or XOD) Detection Kits (Jian Cheng Bioengineering Institute, Nan Jin, Jiangsu, P. R. China), following the manufacturer’s protocol. All the compounds were dissolved in 1% DMSO. The test compounds were initially assayed for their inhibition of AChE and XOD at a concentration of 1.5 mg/mL. If an inhibition of more than 30% was observed, the compound was classified as active. The active compounds were consequently tested at seven concentrations. The results were read on a microplate reader at the wavelength of 450 nm/530 nm. All the assays were performed in triplicate with three independent experiments. The IC_50_ values were calculated using XLfit software.

## 4. Conclusions

In summary, by using active ingredients from *Rhodiola rosea* L. as lead compounds, we adopted three routes to synthesize three categories of natural and synthetic PPGs and tested their AChE and XOD inhibitory activity. Several PPGs were found to have potential inhibitory effects on AChE and XOD which are worthy of further study. This result suggested PPGs may be the active ingredients of *Rhodiola rosea* L. in the therapy of nervous and cardiovascular diseases and also provided some pharmacological basis for the usage of this plant in Traditional Chinese Medicine.

## References

[1] Saratikov A.S., Krasnov E.E., Chnikina L.A., Duvidson L.M., Sotova M.I., Marina T.F., Nechoda M.F., Aksenova R.A., Tschrdinzeff S.G. (1968). Rhodiolosid ein neues Glykosid aus *Rhodiola rosea* und seine pharmakologischen Eigenschaften. Pharmazie.

[2] Brekhman I.I., Dardymov I.V. (1969). New substances of plant origin which increase non-specific resistance. Ann. Rev. Pharmacol..

[3] Sokolov S.Ya., Ivashin V.K., Zapesochnaya G.G., Kurkin V.A., Shchavlinskii A.N. (1985). Studies of neurotropic activity of new compounds isolated from *Rhodiola rosea* L. Khim. Farm. Zh..

[4] Sui R.B., Li X.D., Liu Y.Y., Yang B., Min L.Q. (2006). Interventional effects of gold theragran on plasma levels of cytokine and endothelin in rats with focal cerebral ischemia-reperfusion injury. Chin. J. Clin. Rehabil..

[5] Xu Q., Zhu S.G., Zhou J.W., Kang J.S., Du K.Q. (1999). Study on protection of Rhodiola sachalinensis A.R on cerebral neurons against injury of the ischemia reperfusion in rats. J. Apoplexy Nerv. Dis..

[6] Xu Q., Guo Z.Y., Kang J.S., Zhu S.H., Du K.Q., Yang H.F. (1999). Study on protection of Rhodiola sachalinensis AR against free radical injury of the ischemia reperfusion rats. J. Norman Bethune Univ. Med. Sci..

[7] Liu Y., Zhang X., Ni Z.F., Zhou C.Y., Fan P., Li R.X. (2000). Effects of Nuo Di Kang on myocardial ischemia and blood-lipid and lipoprotein in rats. China Pharm..

[8] Perry E.K. (1986). The cholinergic hypothesis-ten years on. Brit. Med. Bull..

[9] Bartus R.T., Dean R.L., Beer B., Lippa A.S. (1982). The cholinergic hypothesis of geriatric memory dysfunction. Science.

[10] Massey V., Brumby P.E., Komai H., Palmer G. (1969). Studies on milk xanthine oxidase. Some spectral and kinetic properties. J. Biol. Chem..

[11] Cesselli D., Jakoniuk I., Barlucchi L., Beltrami A.P., Hintze T.H., Nadal-Ginard B., Kajstura J., Leri A. (2001). Oxidative stress-mediated cardiac cell death is a major determinant of ventricular dysfunction and failure in dog dilated cardiomyopathy. Circ. Res..

[12] Spiekermann S., Landmesser U., Dikalov S., Bredt M., Gamez G., Tatge H., Reepschläger N., Hornig B., Drexler H., Harrison D.G. (2003). Electron spin resonance characterization of vascular xanthine and NAD(P)H oxidase activity in patients with coronary artery disease: Relation to endothelium-dependent vasodilation. Circulation.

[13] Ulf E G.E., Robert W.H., Ori S., Rajiv N.T., Richard S.T., Hideaki S., David A.K., Eduardo M., Joshua M.H. (1999). Intravenous allopurinol decreases myocardial oxygen consumption and increases mechanical efficiency in dogs with pacing-induced heart failure. Circ. Res..

[14] Walter F.S., Nazareno P., Marcus E.S.J., Michel W.S., Garrick C.S., Xie J.S., Robert W.H., Joshua Z., Daniel M., Eduardo M., David A.K., Joshua M.H. (2002). Imbalance between xanthine oxidase and nitric oxide synthase signaling pathways underlies mechanoenergetic uncoupling in the failing heart. Circ. Res..

[15] Thomas P.C., David A.K., Gregory S.N., Ronald D.B., Gisele O.R., Zoulficar A.K., Eduardo M., Joshua M.H. (2001). Allopurinol improves myocardial efficiency in patients with idiopathic dilated cardiomyopathy. Circulation.

[16] Ute M., Ralf S., Markus P., Bernd L., Manuela B., Andrea P.S., Bernd K., Frank R. (2006). Synthesis of ^131^I-labeled glucose-conjugated inhibitors of O^6^-methylguanine-DNA methyltransferase (MGMT) and comparison with nonconjugated inhibitors as potential tools for *in vivo* MGMT imaging. J. Med. Chem..

[17] Sunil K.C., Oscar H. (1979). A simplified procedure for the preparation of triphenylmethylethers. Tetrahedron Lett..

[18] Akino J., Per J., Bernard B. (1994). Cinnamrutinoses A and B, glycosides of *Populus tremula*. Phytochemistry.

[19] Tolonen A., Pakonen M., Hohtola A., Jalonen J. (2003). Phenylpropanoid glycosides from *Rhodiola rosea*. Chem. Pharm. Bull..

[20] Zapesochnaya G.G., Kurkin V.A. (1982). Glycosides of cinnamyl alcohol from the rhizomes of Rhodiola rosea. Chem. Nat. Compounds.

